# Baicalein Suppresses NLRP3 and AIM2 Inflammasome-Mediated Pyroptosis in Macrophages Infected by Mycobacterium tuberculosis via Induced Autophagy

**DOI:** 10.1128/spectrum.04711-22

**Published:** 2023-05-01

**Authors:** Bangzuo Ning, Jingjing Shen, Fanglin Liu, Hemin Zhang, Xin Jiang

**Affiliations:** a Center for Traditional Chinese Medicine and Immunology Research, School of Basic Medical Sciences, Shanghai University of Traditional Chinese Medicine, Shanghai, China; University of Hawaii at Manoa

**Keywords:** *Mycobacterium tuberculosis*, baicalein, pyroptosis, autophagy, AIM2 inflammasome, NLRP3 inflammasome

## Abstract

Mycobacterium tuberculosis (Mtb) continues to pose a significant threat to global health because it causes granulomas and systemic inflammatory responses during active tuberculosis (TB). Mtb can induce macrophage pyroptosis, which results in the release of IL-1β and causes tissue damage, thereby promoting its spread. In the absence of anti-TB drugs, host-directed therapy (HDT) has been demonstrated to be an effective strategy against TB. In this study, we used an *in vitro* Mtb-infected macrophage model to assess the effect of baicalein, derived from Scutellariae radix, on pyroptosis induced in Mtb-infected macrophages. Further, we investigated the molecular mechanisms underlying the actions of baicalein. The results of the study suggest that baicalein inhibits pyroptosis in Mtb-infected macrophages by downregulating the assembly of AIM2 and NLRP3 inflammasome and promoting autophagy. Further research has also shown that the mechanism by which baicalein promotes autophagy may involve the inhibition of the activation of the Akt/mTOR pathway and the inhibition of the AIM2 protein, which affects the levels of CHMP2A protein required to promote autophagy. Thus, our data show that baicalein can inhibit Mtb infection-induced macrophage pyroptosis and has the potential to be a new adjunctive HDT drug.

**IMPORTANCE** Current strategies for treating drug-resistant tuberculosis have limited efficacy and undesirable side effects; hence, research on new treatments, including innovative medications, is required. Host-directed therapy (HDT) has emerged as a viable strategy for modulating host cell responses in order to enhance protective immunity against infections. Baicalein, extracted from Scutellariae radix, was shown to inhibit pyroptosis caused by Mycobacterium tuberculosis-infected macrophages and was associated with autophagy. Our findings reveal that baicalein can be used as an adjunctive treatment for tuberculosis or other inflammatory diseases by regulating immune function and enhancing the antibacterial ability of the host. It also provides a new idea for exploring the anti-inflammatory mechanism of baicalein.

## INTRODUCTION

Tuberculosis (TB), one of the top 10 causes of death worldwide and the leading cause of death due to a single infectious agent, is a communicable disease. Its causative agent is Mycobacterium tuberculosis (Mtb) (1). TB research and the development of anti-TB medications have been a major cause of concern over the past two decades. The slow-paced development of anti-TB medications and eventual drug resistance to TB are the main issues facing effective drug development (2). Therefore, there is an urgent need for novel TB treatment strategies. Several studies have reported that the primary cause of death in most TB patients is tissue damage caused by an excessive inflammatory response to the infection rather than the bacterial infection itself. As a result, effective TB treatment will require both the elimination of Mtb from the host and the prevention of the inflammatory reaction. Host-directed therapy (HDT) is based on balancing the host immune system and may provide a novel approach to the development of new anti-TB drugs (3). HDT is a unique adjuvant therapy aimed at modulating the protective immune response against pathogens and minimizing excessive inflammation in infected tissues (4). HDT medications can assist the host in reducing the excessive inflammatory response in Mtb-infected macrophages, the key effector cells for the clearance of Mtb, thereby reducing tissue damage and enhancing the treatment efficacy of anti-TB therapies (5, 6).

Pyroptosis is an effective cellular process that helps in the clearing of infected cells. It is a type of lytic cell death that results in the release of a large number of inflammatory cytokines and the destruction of intracellular replicative niches. While this role of pyroptosis is beneficial in case of infection, it can be harmful to the host if it loses control (7, 8). Excessive inflammatory responses in afflicted tissues, leading to severe tissue damage and disease development, is a side effect of pyroptosis (9). Pyroptosis in relation to TB is a double-edged sword, with the negative effect taking precedence. Here, pyroptosis in Mtb-infected macrophages involves the release of proinflammatory mediators such as high mobility group protein (HMGB1) and interleukin-1β (IL-1β) along with the distribution of Mtb leading to the exacerbation of the pathological process and increasing the chances of infection (10, 11).

The canonical pathway of pyroptosis involves multiprotein complexes referred to as inflammasomes, which form in response to external stimuli such as injury, toxins, hypoxia, and pathogens. Sensor, adaptor, and effector caspases are the three main components of inflammasome complex (12). Several members of the NOD-like receptor (NLR) family, including NLRP1, NLRP3, and NLRC4, as well as proteins absent in melanoma 2 (AIM2) and pyrin, have been identified (13, 14). Inflammasome activation promotes the maturation of pro-caspase-1 into caspase-1, which then cleaves immature pro-IL-1β and pro-IL-18 into mature IL-1β and IL-18, respectively. Gasdermin D (GSDMD) is cleaved into N- and C-terminal components by caspase-1. The N-terminal domains bind to cell membranes, forming oligomeric pores and resulting in lytic cell death (15). Inflammasome complexes react to a wide range of pathogens and danger or homeostasis-altering signals, and they can be extremely important in the onset of cancer and autoinflammatory diseases (16). AIM2 is a cytosolic, double-stranded DNA (dsDNA) sensor. When AIM2 detects aberrant cytoplasmic DNA, it combines with the adaptor molecule ASC and inactive pro-caspase-1 to form the AIM2 inflammasome, which stimulates the production of proinflammatory cytokines, such as IL-1β (17, 18). NLRP3 is a well researched inflammasome, and its activation is a crucial initiating factor in pyroptosis. Mtb infection has been shown to promote the activation of NLRP3 and AIM2 inflammasomes and IL-1β production and to increase Mtb burden in adjacent cells (10, 19). Activation of the caspase-1/IL-1β inflammasome is an important first line of defense against microbes (20–23). Mtb can also inhibit inflammasome activation and pyroptosis by using different effectors. Mtb prevents inflammasome activation and IL-1β processing, and a functional Mtb *zmp1* gene is required for this process (24). The serine/threonine kinase PknF of Mtb has been shown to play an important role in innate immune evasion through inhibition of the NLRP3 inflammasome (25). Protein-tyrosine phosphatase B (PtpB) from Mtb alters the phospholipid composition of the host membrane by binding ubiquitin (Ub) to inhibit pyroptosis and counteract host immunity. PtpB is a potent inhibitor of host pyroptosis that subverts GSDMD functions to facilitate Mtb intracellular survival (26). Mtb Rv3364c protein can inhibit caspase-1 activation and pyroptosis by interacting with macrophage membrane-associated serine protease and cathepsin G and inhibiting its enzymatic activity (27).

Autophagy modulates pyroptosis, and according to a 2008 study, loss of an autophagy-related protein (ATG) increases IL-1β release and cell lysis following pyroptosis (28). The interaction between these two cellular events drew the attention of scientists. Autophagy is a self-degradation and self-maintenance process in eukaryotic cells that plays a crucial role in removing damaged organelles, proteins, and cell fragments (29). During this process, pathogens, abnormal proteins, and organelles are bilayer wrapped to create autophagosomes, which are then transported to lysosomes for degradation (30). Autophagy negatively regulates pyroptosis. It inhibits pyroptosis first by eliminating pathogen-associated molecular patterns (PAMPs) and damage-associated molecular patterns (DAMPs) and second by inhibiting its fundamental components (13, 31, 32).

Baicalein (5,6,7-trihydroxyflavone) is a flavonoid derived from the root of Scutellariae radix. Several studies have demonstrated a wide range of pharmacological effects related to its actions in preventing cancer, inflammation, pathogenic infections, and oxidation (33, 34). The anti-inflammatory effects of baicalein have been extensively discussed in previous researches (35–38). However, further research is required to determine the role of baicalein in Mtb infections. This study aimed to investigate the effect of baicalein on AIM2 and NLRP3 inflammasome activation and subsequent pyroptosis following Mtb infection. We found that the inhibition of pyroptosis by baicalein may be related to autophagy.

## RESULTS

### Effect of baicalein on the viability of J774A.1 and U937 cells.

**FIG 1:**
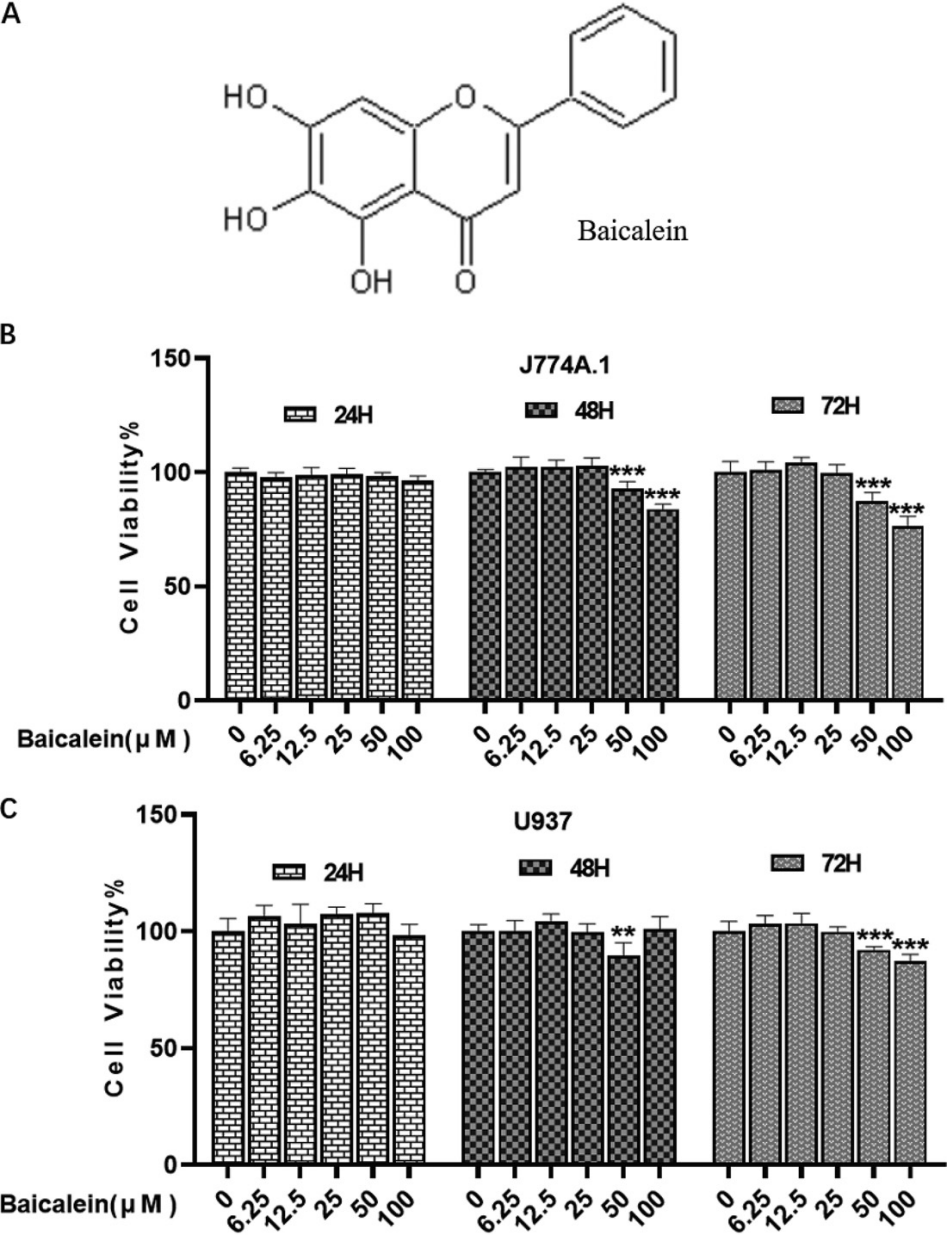
Effect of baicalein on the viability of J774A.1 and U937 cells. (A) The chemical structure of baicalein. (B) The viability of J774A.1 cells that were treated by different concentrations of baicalein at different time points was assessed by viability assay (data compared with control). (C) The viability of U937 cells that were treated by different concentrations of baicalein at different time points was assessed by viability assay (data compared with control). The data are shown as means ± standard deviation (SD) of at least three independent experiments. **, *P* values (*P*) < 0.01; ***, *P < *0.001.

To exclude cellular toxicity and optimize the concentration of baicalein (Bai), the viability of J774A.1 and U937 cells treated with baicalein were assessed by performing the viability assay. The results showed that baicalein (6.25, 12.5, 25, 50, and 100 μM) did not affect cell viability at 24 h, and baicalein (50 and 100 μM) reduced cell viability at 48 and 72 h (Fig. 1B and C). In the follow-up experiment, the treatment time of baicalein (25, 50, and 100 μM) should not exceed 24 h.

### Baicalein inhibited pyroptosis in Mtb-infected J774A.1 and U937 cells.

**FIG 2:**
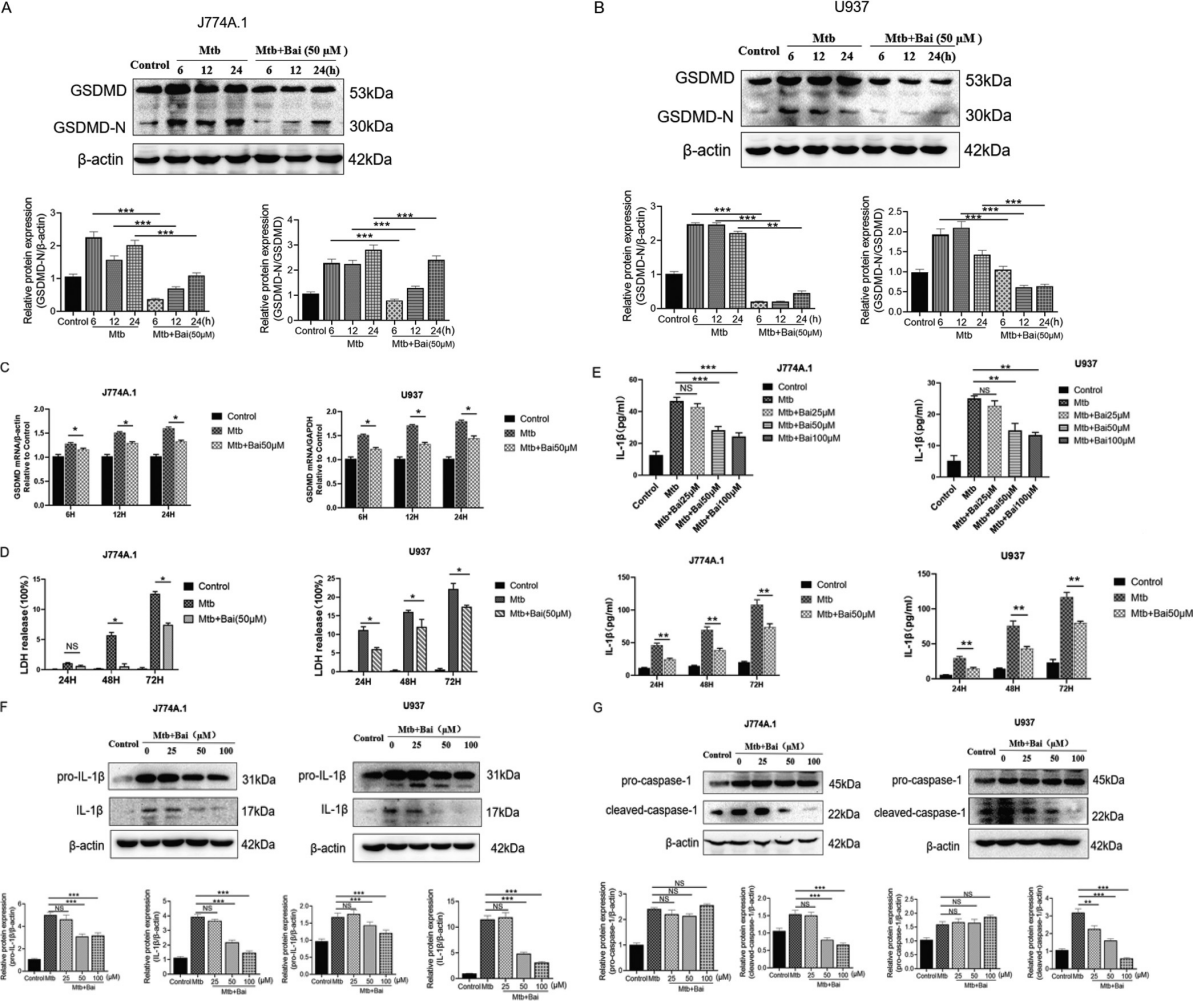
Baicalein (Bai) inhibited pyroptosis in M. tuberculosis (Mtb)-infected J774A.1 and U937 cells. (A) Levels of gasdermin D (GSDMD)-N protein were detected by Western blotting in J774A.1 cells, the bar graphs show the relative intensity of GSDMD-N. (B) Levels of GSDMD-N protein were detected by Western blotting in U937 cells. The bar graphs show the relative intensity of GSDMD-N. (C) Levels of GSDMD mRNA were investigated by real-time PCR in J774A.1 and U937 cells, respectively. (D) Cell death was detected by using the lactate dehydrogenase (LDH) cytotoxicity assay kit in J774A.1 and U937 cells, respectively. (E) Levels of interleukin 1β (IL-1β) determined by enzyme-linked immunosorbent assay (ELISA) measurement in J774A.1 and U937 cells, respectively. (F) Levels of pro-IL-1β and IL-1β proteins were detected by Western blotting in J774A.1 and U937 cells. The bar graphs show the relative intensity of pro-IL-1β and IL-1β. (G) Levels of pro-caspase-1 and cleaved-caspase-1 proteins were detected by Western blotting in J774A.1 and U937 cells. The bar graphs show the relative intensity of pro-caspase-1 and cleaved-caspase-1. The data are shown as means ± SD of three independent experiments. *, *P < *0.05; **, *P < *0.01; ***, *P < *0.001. NS, nonsignificant.

Given the role of pyroptosis in inflammation, we hypothesized that baicalein may suppress pyroptosis in Mtb-infected macrophages to reduce inflammation in the case of TB. In this study, J774A.1 and U937 cells were infected with Mtb to generate an appropriate set of experimental models. Based on the previous viability assay, we treated Mtb-infected macrophages with baicalein (50 μM) for different times (6, 12, and 24 h). After collecting samples, the levels of GSDMD protein were detected by Western blotting. The results showed that while N-terminal of GSDMD (GSDMD-N) protein levels increased in J774A.1 and U937 cells infected with Mtb, baicalein therapy decreased GSDMD-N protein levels (Fig. 2A and B). To determine whether baicalein affects the GSDMD expression levels in the Mtb infection model, the mRNA of GSDMD was detected in the Mtb group and the baicalein group. The results showed that baicalein (50 μM) could inhibit the expression of GSDMD gene (Fig. 2C). During pyroptosis, the relatively stable enzyme lactate dehydrogenase (LDH) is released from the cells, and its activity can be used to demonstrate the integrity of the cell membrane. Mtb infection can enhance the production of LDH at various time intervals; however, baicalein (50 μM) can reverse this effect in J774A.1 and U937 cells within all time points (Fig. 2D). During pyroptosis, Mtb-infected macrophages produce IL-1β, which is released from the cell membrane, resulting in an inflammatory reaction. We treated Mtb-infected macrophages with different concentrations of baicalein (25, 50, and 100 μM) for 24 h to detect the content of IL-1β. The results showed that 50 and 100 μM baicalein could inhibit the expression of IL-1β. We treated Mtb-infected macrophages with 50 μM baicalein for 24, 48, and 72 h to detect the content of IL-1β. The results showed that baicalein (50 μM) significantly inhibited the expression of IL-1β at 48 and 72 h (Fig. 2E). We treated Mtb-infected macrophages with baicalein (25, 50, and 100 μM) for 12 h and subsequently examined the levels of pro-IL-1β and IL-1β proteins by Western blotting. The results showed that 50 and 100 μM baicalein treatment significantly decreased pro-IL-1β and IL-1β protein levels (Fig. 2F). Caspase-1 is activated during pyroptosis. The results showed that in J774A.1 and U937 cells infected with Mtb, the protein levels of pro-caspase-1 and cleaved-caspase-1 increased, and after treatment with baicalein, the protein levels of cleaved-caspase-1 decreased significantly, while that of pro-caspase-1 protein levels had no effect (Fig. 2G). These results demonstrated that baicalein treatment ameliorates pyroptosis in Mtb-infected macrophages.

### Baicalein inhibits Mtb-induced pyroptosis associated with NLRP3 and AIM2 inflammasomes.

**FIG 3:**
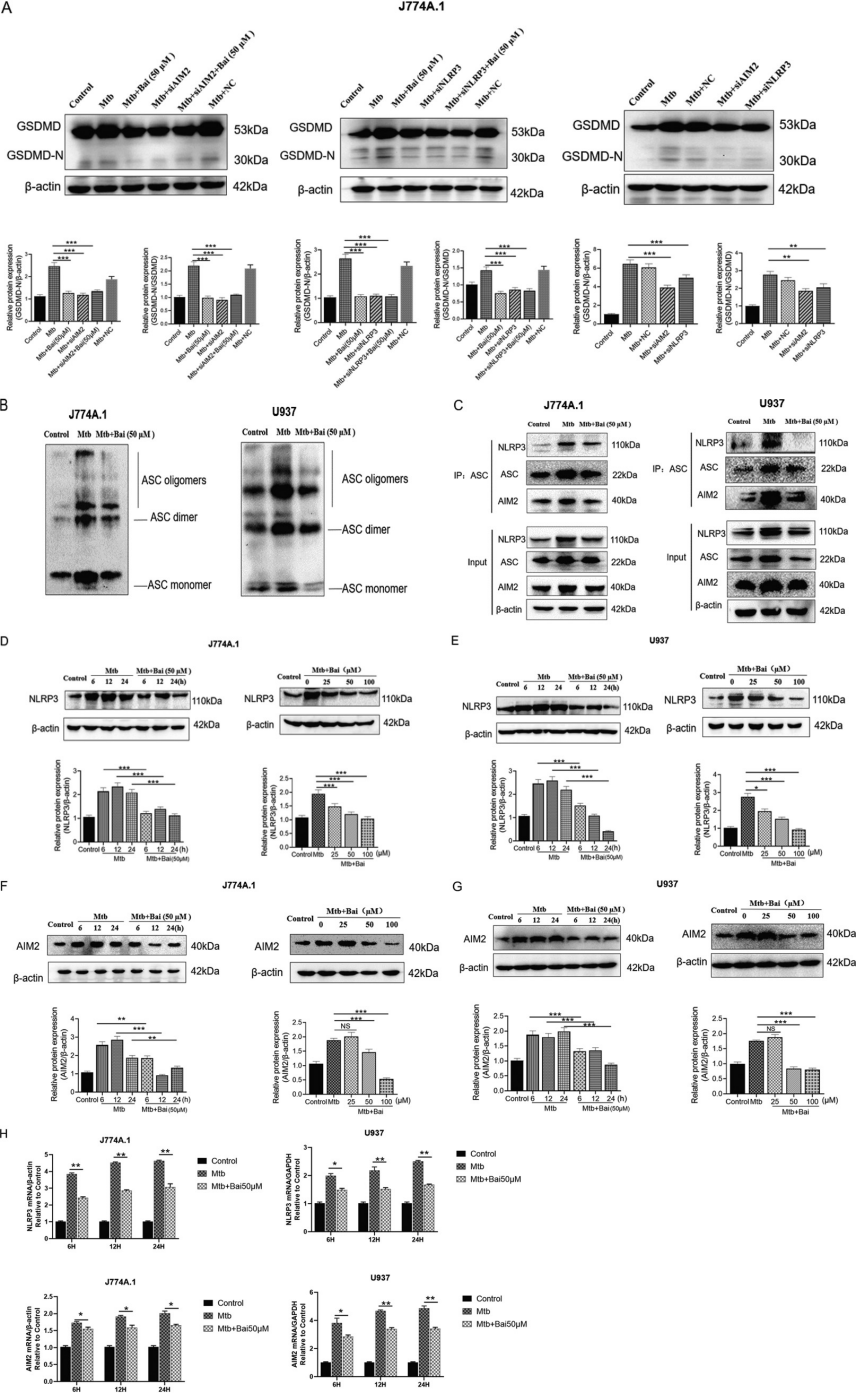
Baicalein inhibits Mtb-induced pyroptosis associated with NLRP3 and AIM2 inflammasomes. (A) Levels of AIM2 protein were detected by Western blotting after small interfering RNAs (siRNAs) of AIM2 and NLRP3 in J774A.1 cells. The bar graphs show the relative intensity of GSDMD-N. (B) Western blotting analysis for ASC in Triton X-100-insoluble pellets cross-linked with disuccinimidyl suberate in J774A.1 and U937 cells, respectively. (C) ASC coimmunoprecipitation (co-IP) from J774A.1 and U937 cells were immunoblotted for NLRP3, ASC, and AIM2 and reblotted for NLRP3, ASC, and AIM2, respectively. (D) Levels of NLRP3 protein were detected by Western blotting in J774A.1 cells. The bar graphs show the relative intensity of NLRP3. (E) Levels of NLRP3 protein were detected by Western blotting in U937 cells. The bar graphs show the relative intensity of NLRP3. (F) Levels of AIM2 protein were detected by Western blotting in J774A.1 cells. The bar graphs show the relative intensity of AIM2. (G) Levels of AIM2 protein were detected by Western blotting in U937 cells. The bar graphs show the relative intensity of AIM2. (H) Levels of NLRP3 and AIM2 mRNA were investigated by real-time PCR in J774A.1 and U937 cells, respectively. The data are shown as means ± SD of three independent experiments. NC, negative control. *, *P < *0.05; **, *P < *0.01; ***, *P < *0.001. NS, nonsignificant.

To determine whether AIM2 and NLRP3 inflammasomes are involved in Mtb-induced pyroptosis, GSDMD-N protein levels were assessed after transfection with AIM2 and NLRP3 small interfering RNAs (siRNAs) for 36 h. The results showed that GSDMD-N protein levels decreased in Mtb-infected J774A.1 cells after transfection with AIM2 siRNA and NLRP3 siRNA, suggesting that both AIM2 and NLRP3 proteins are involved in the induction of pyroptosis (Fig. 3A). Compared with siAIM2/siNLRP3, baicalein (50 μM) combined with siAIM2/siNLRP3 did not show the additive effect of reducing the protein levels of GSDMD-N, which may be due to the fact that baicalein can also inhibit the protein levels of AIM2/NLRP3, which has the same effect as siAIM2/siNLPR3. Activation of the inflammasome induces supramolecular oligomerization of ASC dimers into massive intertwining fibrils, commonly known as “ASC specks” or “pyroptosomes” (39). ASC speck/pyroptosomes are associated with caspase-1 cleavage and mature IL-1β release (40). Infection of J774A.1 and U937 cells with Mtb led to the emergence of immunoreactive bands for ASC oligomers at molecular weights corresponding to ASC monomers, dimers, and higher-order oligomers. The ASC oligomers in the baicalein (50 μM) treatment group were less than those in the Mtb infection group at 12h (Fig. 3B). Coimmunoprecipitation (co-IP) experiments utilizing an ASC antibody revealed that the interactions between NLRP3 and ASC, as well as between AIM2 and ASC, were significantly enhanced in Mtb-infected cells. Compared with the Mtb infection group, after baicalein (50 μM) treatment for 12 h, the protein levels of NLPR3, ASC, and AIM2 decreased, the interactions between NLRP3 and ASC, as well as between AIM2 and ASC, were decreased (Fig. 3C). The results showed that NLRP3 and AIM2 protein levels increased following Mtb infection; however, these effects were significantly reversed when treated with baicalein at various concentrations and durations (Fig. 3D to G). To determine whether baicalein (50 μM) has an effect on the NLRP3 and AIM2 expression levels in the Mtb infection model, the mRNA of NLRP3 and AIM2 were detected in the Mtb group and the baicalein group. The results showed that baicalein could inhibit the expression of NLRP3 and AIM2 gene (Fig. 3H). This implies that Mtb infection may be able to induce the activation of NLRP3 and AIM2 inflammasomes, leading to pyroptosis. Hence, inhibition of pyroptosis by baicalein in Mtb-infected cells is associated with inhibition of NLRP3 and AIM2 inflammasome activation.

### Baicalein inhibition of pyroptosis is associated with autophagy.

**FIG 4:**
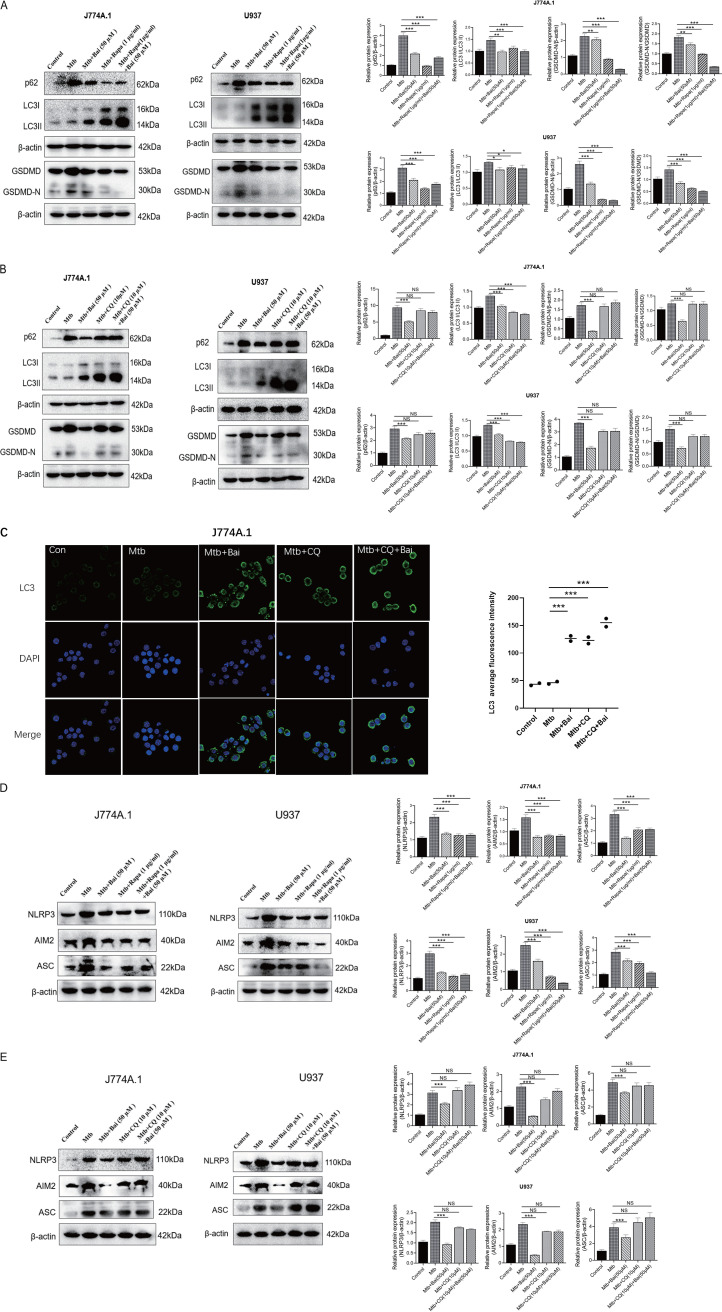
Baicalein promotes autophagy and inhibits pyroptosis in Mtb-infected macrophages. (A) Levels of p62, LC3 II, GSDMD, and GSDMD-N proteins after baicalein and rapamycin treatment were detected by Western blotting in J774A.1 and U937 cells. The bar graphs show the relative intensity of p62, LC3II, and GSDMD-N. (B) Levels of p62, LC3 II, GSDMD, and GSDMD-N proteins after baicalein and CQ treatment were detected by Western blotting in J774A.1 and U937 cells. The bar graphs show the relative intensity of p62, LC3II, and GSDMD-N. (C) Rabbit anti-LC3 (green) and 4′,6-diamidino-2-phenylindole (DAPI; blue) were used for immunofluorescence staining. The fluorescence signal and localization of LC3 in J774A.1 cells were observed by confocal microscopy. (D) Levels of NLRP3, AIM2, and ASC proteins after baicalein and rapamycin treatment were detected by Western blotting in J774A.1 and U937 cells. The bar graphs show the relative intensity of NLRP3, AIM2, and ASC. (E) Levels of NLRP3, AIM2, and ASC proteins after baicalein and CQ treatment were detected by Western blotting in J774A.1 and U937 cells. The bar graphs show the relative intensity of NLRP3, AIM2, and ASC. The data are shown as means ± SD of three independent experiments. *, *P < *0.05; **, *P < *0.01; ***, *P < *0.001. NS, nonsignificant.

It has been proven that autophagy inhibits caspase-1-dependent pyroptosis (41). The formation of microtubule-associated protein 1A/1B-light chain 3 II (LC3) II is a hallmark of autophagy. The degradation of p62, an autophagosome substrate protein, marks the formation of highly lytic degradative organelle, the autolysosome, which results from the union of the autophagosome and lysosome. Rapamycin, a known inhibitor of mammalian target of rapamycin (mTOR), can induce autophagy, whereas chloroquine (CQ) is an autophagy inhibitor. To evaluate the effect of autophagy on pyroptosis of J774A.1 and U937 cells after Mtb infection, Mtb-infected cells were treated with rapamycin (1 μg/mL), CQ (10 μM), and baicalein (50 μM) for 12 h, samples were collected for Western blotting to detect the levels of LC3 II, p62, and GSDMD-N proteins. When compared with rapamycin treatment, treatment with baicalein also reduced the levels of p62 protein in J774A.1 and U937 cells following Mtb infection and promoted LC3 II protein levels. This indicates that baicalein promotes autophagy. However, both rapamycin and baicalein treatment reduced the levels of GSDMD-N protein, a key protein in pyroptosis (Fig. 4A). Both baicalein (50 μM) and rapamycin (1 μg/mL) can promote effective autophagy, but the combined use did not see an additive effect. This may be due to the similar effect of the two, which cannot produce superimposed effects. To further validate the efficacy of baicalein in autophagy and pyroptosis, we assessed protein levels changes following the use of the autophagy inhibitor CQ. As shown, CQ caused significant accumulation of LC3 II and p62 protein levels, and the level of GSDMD-N protein was evident (Fig. 4B). This indicated a blockage in the effects of baicalein in promoting autophagy and inhibiting pyroptosis. Correspondingly, immunofluorescence assays revealed that baicalein (50 μM) and CQ (10 μM) promoted the accumulation of LC3 on cells after Mtb infection (Fig. 4C). Although the results of different cell lines responding to the same drug are not exactly the same, it may indicate that baicalein inhibits pyroptosis by promoting autophagy.

Under physiological conditions, autophagy is often maintained at a minimal level. The elimination of aberrant proteins from cells to support cell viability through autophagy can be significantly increased by stress (42). To determine whether autophagy has inhibitory effects on AIM2 and NLRP3 inflammasomes, we examined the levels of AIM2, ASC, and NLRP3 proteins. As shown, the levels of AIM2, NLRP3, and ASC proteins in Mtb-infected J774A.1 and U937 cells decreased with rapamycin and baicalein treatment (Fig. 4D). Following the treatment with CQ, inhibitory effect of baicalein on AIM2, ASC, and NLRP3 protein levels also disappeared (Fig. 4E). Although the results of different cell lines treated with the same drug were not exactly the same, the results may indicate that both baicalein and rapamycin inhibited the expression of inflammasome-related proteins by promoting autophagy to degrade activated inflammasomes. After blocking autophagy with CQ, the inhibitory effect of baicalein on inflammasome-associated proteins was also blocked. It shows that baicalein inhibits the expression of inflammasome-associated proteins and is related to autophagy. In summary, we used rapamycin and CQ to compare baicalein treatment, and the results suggest that the effect of baicalein on inhibiting pyroptosis was related to promoting autophagy.

### Baicalein activates autophagy by inhibiting Akt/mTOR signaling pathway.

**FIG 5:**
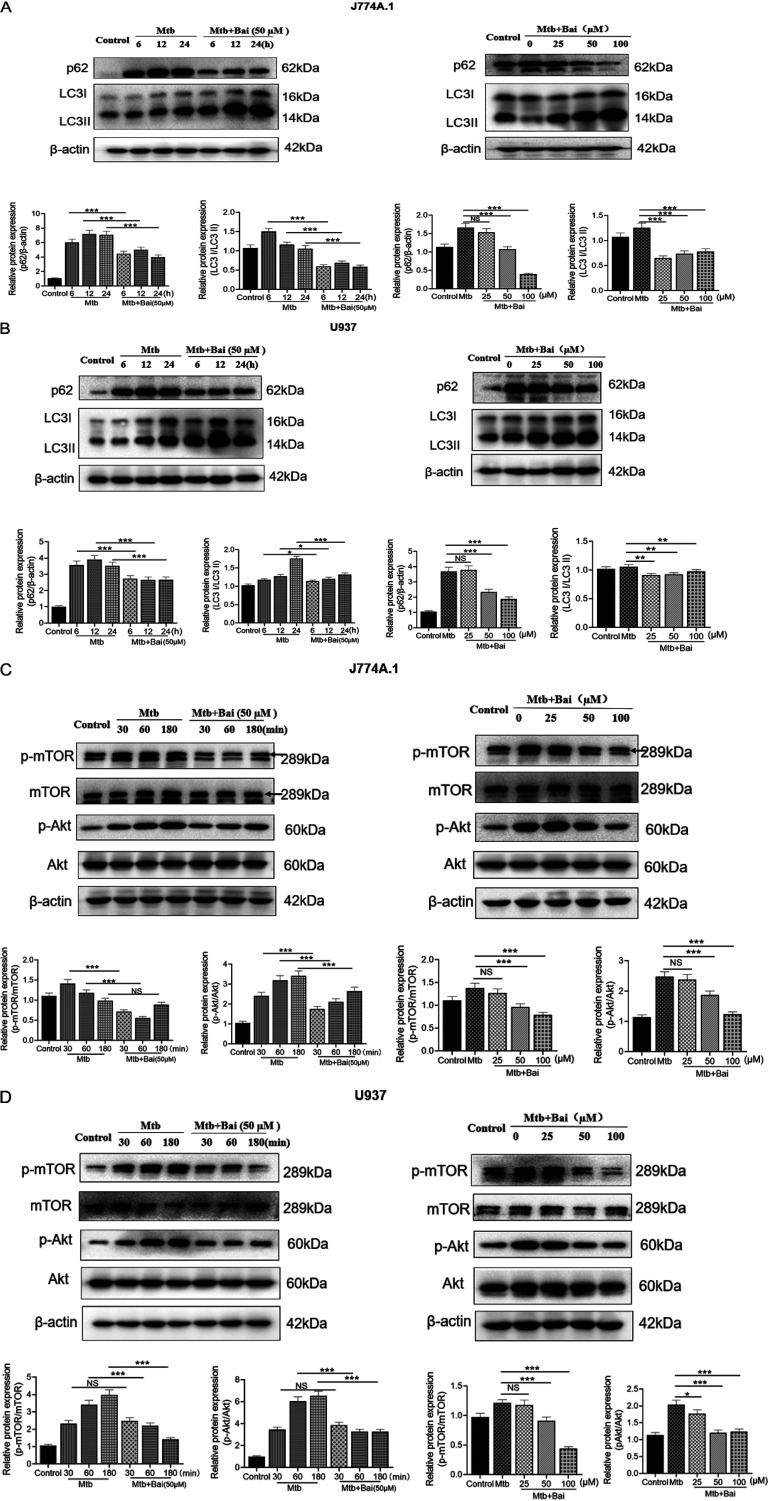
Baicalein activates autophagy by inhibiting Akt/mTOR signaling pathway. (A) Levels of p62 and LC3II proteins were detected by Western blotting in J774A.1 cells. The bar graphs show the relative intensity of p62, LC3II. (B) Levels of p62 and LC3II proteins were detected by Western blotting in U937 cells. The bar graphs show the relative intensity of p62 and LC3II. (C) Levels of p-Akt and p-mTOR proteins were detected by Western blotting in J774A.1 cells. The bar graphs show the relative intensity of p-Akt and p-mTOR. Total mTOR and Akt were used as an internal control. (D) Levels of p-Akt and p-mTOR proteins were detected by Western blotting in U937 cells. The bar graphs show the relative intensity of p-Akt and p-mTOR. Total mTOR and Akt were used as an internal control. The data are shown as means ± SD of three independent experiments. *, *P < *0.05; **, *P < *0.01; ***, *P < *0.001. NS, nonsignificant.

To evaluate the effect of baicalein on autophagy induction, we examined the levels of LC3 II and p62 proteins after treatment of Mtb-infected macrophages with different concentrations of baicalein for 12 h. As shown in Fig. 5A and B, baicalein induced the activation of autophagy, with concentrations of 50 and 100 μM triggering the most efficient autophagy. Baicalein also induced effective levels of autophagy at 6, 12, and 24 h (Fig. 5A and B). Activation of autophagy can be influenced by a wide range of signals. For instance, inhibition of the Akt/mTOR pathway induces autophagy activation. Hence, we analyzed the effect of baicalein on Akt/mTOR signaling pathways. The results showed that baicalein (50 μM) treatment inhibited the phosphorylation of Akt and mTOR in J774A.1 and U937 cells at some time points. Moreover, baicalein (50 and 100 μM) inhibited Akt and mTOR phosphorylation in J774A.1 and U937 cells at 60 min (Fig. 5C and D). Thus, our data revealed that baicalein inhibited the Akt/mTOR signaling pathway to activate autophagy.

### Reduction of AIM2 rather than NLRP3 contributes to autophagy activation.

**FIG 6:**
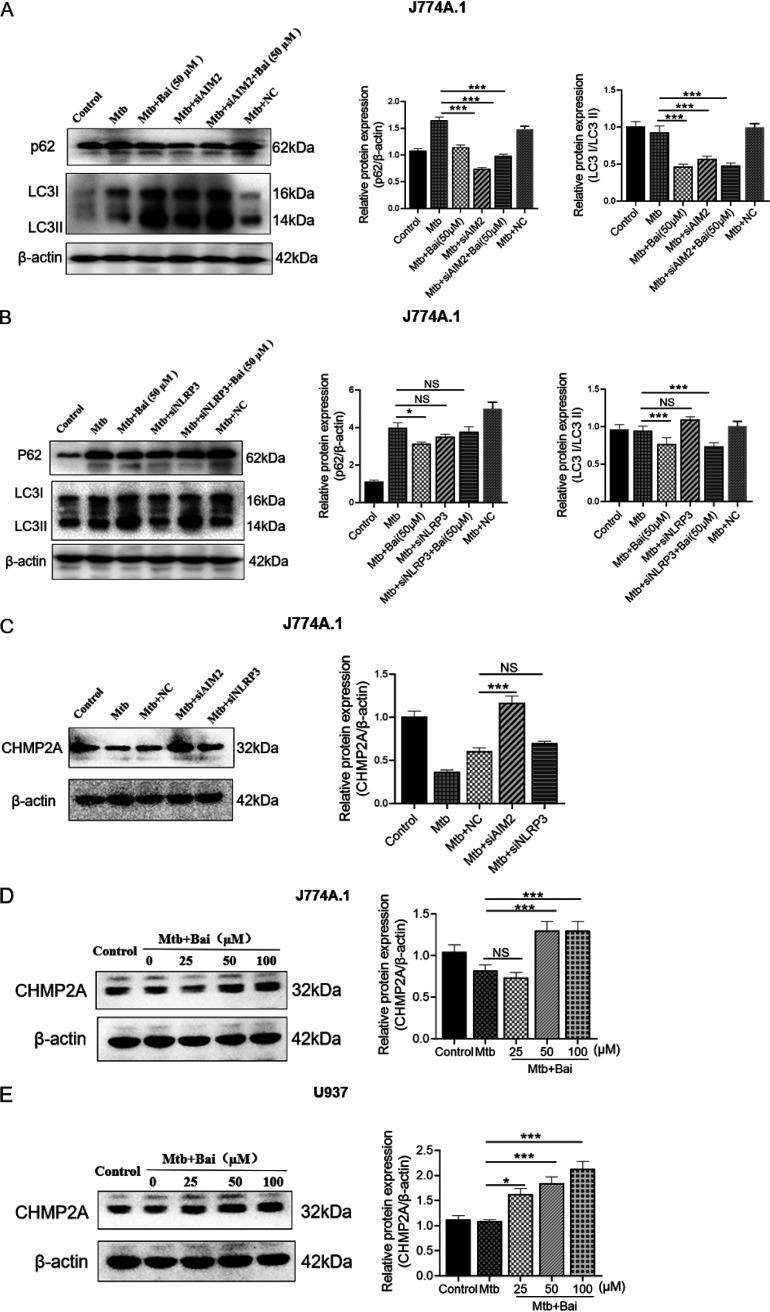
A reduction in AIM2 contributes to autophagy activation but not NLRP3. (A) Levels of p62 and LC3 II proteins were detected by Western blotting after siRNAs of AIM2 in J774A.1 cells. The bar graphs show the relative intensity of p62 and LC3 II. (B) Levels of p62 and LC3 II proteins were detected by Western blotting after siRNAs of NLRP3 in J774A.1 cells. The bar graphs show the relative intensity of p62, LC3 II. (C) Levels of CHMP2A protein were detected by Western blotting after siRNAs of AIM2 and NLRP3 in J774A.1 cells, The bar graphs show the relative intensity of CHMP2A. (D) Levels of CHMP2A protein were detected by Western blotting in J774A.1 cells. The bar graphs show the relative intensity of CHMP2A. (E) Levels of CHMP2A protein were detected by Western blotting in U937 cells. The bar graphs show the relative intensity of CHMP2A. The data are shown as means ± SD of three independent experiments. *, *P < *0.05; **, *P < *0.01; ***, *P < *0.001. NC, negative control; NS, nonsignificant.

Previous studies have demonstrated that autophagy may regulate inflammasomes (43, 44). However, their relationship is complex. To better understand whether AIM2 and NLRP3 proteins can affect autophagy after Mtb infection, we transfected J774A.1 cells with AIM2/NLRP3 siRNA for 36 h and observed the levels of autophagy-related proteins p62 and LC3 II. The results showed that the level of p62 protein decreased and that of LC3 II protein increased in Mtb-infected J774A.1 cells after transfection with AIM2 siRNA, indicating that interfering with AIM2 protein can induce autophagy (Fig. 6A). Since baicalein can both promote autophagy and inhibit the levels of AIM2 protein, the treatment of baicalein (50 μM) combined with siAIM2 did not see the superimposed effect. In contrast, the levels of p62 and LC3 II proteins in Mtb-infected J774A.1 cells was not statistically different after transfection with NLRP3 siRNA, indicating that NLRP3 protein does not affect autophagy (Fig. 6B).

Mtb is an intracellular pathogen that can multiply within infected macrophages by inhibiting the development of the phagosomes and autophagosomes in its presence. However, its mechanism of action remains unclear. Furthermore, considerably less is known about the late stages of autophagy, particularly autophagosome maturation, during which the double-membrane seals contain the contents and eventually fuse with the lysosome (45). Recently, it was shown that the endosomal sorting complex required for transport (ESCRT) III component charged multivesicular body protein 2A (CHMP2A) regulates phagophore closure (46, 47). CHMP2A depletion causes autophagosome accumulation and cell death (45). Recent studies have shown that AIM2-related increase in inflammation is associated with reduced CHMP2A levels and impaired autophagy, which are strongly associated with the disease (48). Transfection of AIM2 siRNA and NLRP3 siRNA had different effects on autophagy in Mtb-infected J774A.1 cells; however, it was unclear whether this was due to CHMP2A protein. The results suggested that in J774A.1 cells, the levels of CHMP2A protein in the transfected AIM2 siRNA group was higher than that in the NC group, but there was no statistical significance after transfected with NLRP3 siRNA (Fig. 6C). AIM2 siRNA promoted autophagy may be related to the CHMP2A protein. The levels of CHMP2A in Mtb-infected J774A.1 and U937 cells treated with baicalein were detected by Western blotting. We found that baicalein (50 μM) could upregulate the levels of CHMP2A protein at 12 h of treatment (Fig. 6D and E). This suggests that an alternate mechanism by which baicalein promotes autophagy may be promoting the levels of CHMP2A protein by inhibiting AIM2 but not NLRP3.

## DISCUSSION

Current anti-TB drugs seek to eradicate Mtb infection. However, the long-term use of anti-TB drugs can result in various degrees of toxic side effects and poor patient compliance, which facilitates the development of drug resistance. These challenges prompted the development of a novel therapeutic strategy, HDT, which aims to either directly improve the host’s capacity to fight off microbes or to reduce collateral tissue damage caused by excessive inflammation (49). Researchers are looking into small molecule HDT candidates that target immune cells’ antimicrobial and inflammatory response functions. Baicalein, one of the most abundant flavonoids in *Scutellariae radix*, has broad pharmacological effects in the prevention of cancer, inflammation, pathogen infection, and oxidation (35, 50). Previous studies have covered the anti-inflammatory properties of baicalein in great detail (51, 52). Pyroptosis, which was described recently, is a type of cell death that, unlike apoptosis, may induce excessive inflammatory responses (53). According to previous research, Mtb infection can cause macrophage pyroptosis, which results in the release of proinflammatory molecules and worsens tissue damage. Pyroptosis in Mtb-infected macrophages can result in systemic Mtb release and enhanced infection (10). Based on the inhibitory effect of HDT on excessive inflammation, this study found that baicalein inhibited Mtb infection-induced pyroptosis and its possible mechanism.

In this study, the effect of baicalein on pyroptosis was evaluated using Mtb-infected mouse and human macrophage models *in vitro*. Currently, pyroptosis is divided into canonical and noncanonical pathways. Caspase-1 proteolytically cleaves the precursors of IL-1β and IL-18, as well as the pyroptosis inducer GSDMD, which triggers pyroptosis via the canonical pyroptosis pathway. The noncanonical pyroptosis pathway is also called the caspase-1-independent inflammasome pathway because the cleavage of GSDMD is not mediated by caspase-1 but via caspase-4/5/11 (54). Therefore, we anticipated baicalein to prevent excessive inflammatory responses by suppressing pyroptosis in Mtb-infected macrophages. In this study, we used Western blotting to assess GSDMD-N and cleaved-caspase-1 protein levels to prove that baicalein treatment inhibits pyroptosis in Mtb-infected macrophages. To determine whether baicalein inhibits Mtb-infected macrophage pyroptosis related to canonical or noncanonical pyroptosis pathways, we used Western blotting to identify mouse caspase-11 protein levels in J774A.1 cells. We discovered that following Mtb infection, caspase-11 was activated, indicating that noncanonical pyroptosis occurred simultaneously (data not shown). However, baicalein treatment had no effect on caspase-11 protein levels (data not shown). Therefore, we concluded that the inhibition of pyroptosis by baicalein is mostly due to the inhibition of canonical pathway.

Many studies have found that baicalein is a specific inhibitor of 12/15-lipoxygenase (55–57). Does the inhibitory effect of baicalein on lipoxygenase play a role in this study? We also transfected J774A.1 cells with 12-lipoxygenase (LOX) siRNA for 36 h; then Mtb was infected and treated with baicalein (50 μM) for 12 h, and we found that transfection of 12-LOX siRNA did not affect the levels of GSDMD-N, p62, and LC3 proteins after Mtb infection, while baicalein treatment increased autophagy levels (decreased p62 protein levels, increased LC3II protein levels) and inhibited pyroptosis (decreased GSDMD-N protein levels) (data not shown). Therefore, the results suggest that the effect of baicalein on inhibiting pyroptosis and promoting autophagy in Mtb-infected macrophages has nothing to do with baicalein being a 12/15-lipoxygenase inhibitor. What is the effect of baicalein on cytokines other than IL-1β? Does baicalein induce anti-inflammatory cytokines and inhibit proinflammatory cytokines? We detected anti-inflammatory cytokines (IL-10) and proinflammatory cytokines (tumor necrosis factor α [TNF-α] and IL-6) using quantitative PCR (qPCR). The results suggest that baicalein can inhibit the expression of TNF-α and IL-6 and promote the expression of IL-10 (data not shown). We also detected the effect of NF-κB pp65 protein levels and found that baicalein could inhibit the levels of NF-κB pp65 protein (data not shown). Baicalein has a wide range of effects, and the effects of baicalein may affect cells through multiple pathways and multiple targets.

Pyroptosis is accompanied by many factors, including inflammasome activation (9, 11). GSDMD-N protein levels were assessed after transfection with AIM2 siRNA and NLRP3 siRNA, and the results indicated that both have similar effects on pyroptosis, suggesting that both AIM2 and NLRP3 inflammasomes are involved in Mtb-induced pyroptosis. The co-IP and ASC oligomer cross-linking results indicated that baicalein downregulates the assembly of AIM2 and NLRP3 inflammasomes, possibly by inhibiting AIM2 and NLRP3 protein levels. It has been shown that autophagy negatively regulates pyroptosis (13). We investigated whether the inhibitory effect of baicalein on pyroptosis is related to autophagy by treating Mtb-infected macrophages with rapamycin and CQ. Interestingly, pharmacological stimulation or inhibition of autophagy influences AIM2 and NLRP3 inflammasome activation *in vitro*, implying that autophagy is involved in inflammasome clearance. Baicalein inhibits pyroptosis by promoting autophagic degradation of AIM2 and NLRP3 inflammasomes. The most important pathway involved in autophagy activation is Akt/mTOR. Our study proved that baicalein could inhibit the activation of the Akt/mTOR pathway following Mtb infection.

However, researchers have not yet determined whether inflammation is the result or the cause of impaired autophagic flux. When autophagy is inhibited, IL-1β production increases, and inflammasomes are activated (58). In contrast, AIM2 and NLRP3 inflammasome activation induces caspase-1 activation, which inhibits mitophagy and amplifies mitochondrial damage and pyroptotic cell death (59). Based on these findings, we hypothesized that AIM2 and NLRP3 negatively regulate autophagy. Under normal conditions, decreased AIM2 protein levels had no effect on LC3 protein levels. Following the induction of infection in J774A.1 by Mtb, silencing of AIM2 restored autophagic flux, with a significant increase in LC3 II and a rapid decrease in p62 protein levels. However, silencing of NLRP3 did not produce similar results. These results suggest that inhibiting AIM2 protein triggers autophagy, which may be linked to an increase in CHMP2A protein levels. Numerous Mtb proteins and lipid effectors are thought to contribute to the arrest of phagosome maturation, which is a complex, multistep process (60, 61). Several studies have shown that the loss of CHMP2A leads to the accumulation and blockage of immature autophagosomal structures (62). It has been confirmed that oxygen-glucose deprivation and reperfusion (OGD-Rep)-induced AIM2 inflammasome activation is inhibited by CHMP2A-mediated autophagy. Therefore, impaired autophagic flux decreases autophagic degradation of AIM2 and NLRP3 inflammasome and increases IL-1β release, resulting in pyroptosis in Mtb-infected cells. However, baicalein effectively restores autophagy, promotes the degradation of AIM2 and NLRP3 inflammasomes, and inhibits Mtb-induced pyroptosis. Interestingly, restoring CHMP2A levels or inhibiting AIM2, but not NLRP3, could restore autophagic flux, which is another mechanism by which baicalein promotes autophagy.

This study has some limitations. The involvement of AIM2 and NLRP3 inflammasomes in Mtb infection-induced pyroptosis has been demonstrated. However, other inflammasomes may also be involved, which will be the focus of future studies. Additionally, we used H37Ra strain to infect J774A.1 and U937 cells as *in vitro* infection models. H37Ra is an avirulent strain with a genetic background comparable to that of H37Rv strain; however, it is easier and safer to study (63, 64). The H37Ra strain has a defunct PhoP/R system that affects the virulence mechanism, while H37Rv infection induces a more severe inflammatory immune response associated with tissue damage (65, 66). In contrast to H37Ra, H37Rv infection showed a richer transcriptional pattern of virulence factors that were linked with severe inflammatory immune response and altered metabolic pattern (67). The bacterial secretion system and the transmembrane transport system may be important determinants of the ability of distinct Mtb strains to cause disease (68). Recent studies have found that H37Rv infection induces significantly higher levels of pyroptosis rather than apoptosis in macrophages. H37Rv infection in macrophages induced higher levels of IL-1β and cleaved-caspase-1 expression than H37Ra, implying that Mtb-induced pyroptosis is related to the virulence of Mtb strains (69). The effect of baicalein in inhibiting pyroptosis in this study can also be referred to in the future study of pyroptosis induced by H37Rv strain infection.

**FIG 7:**
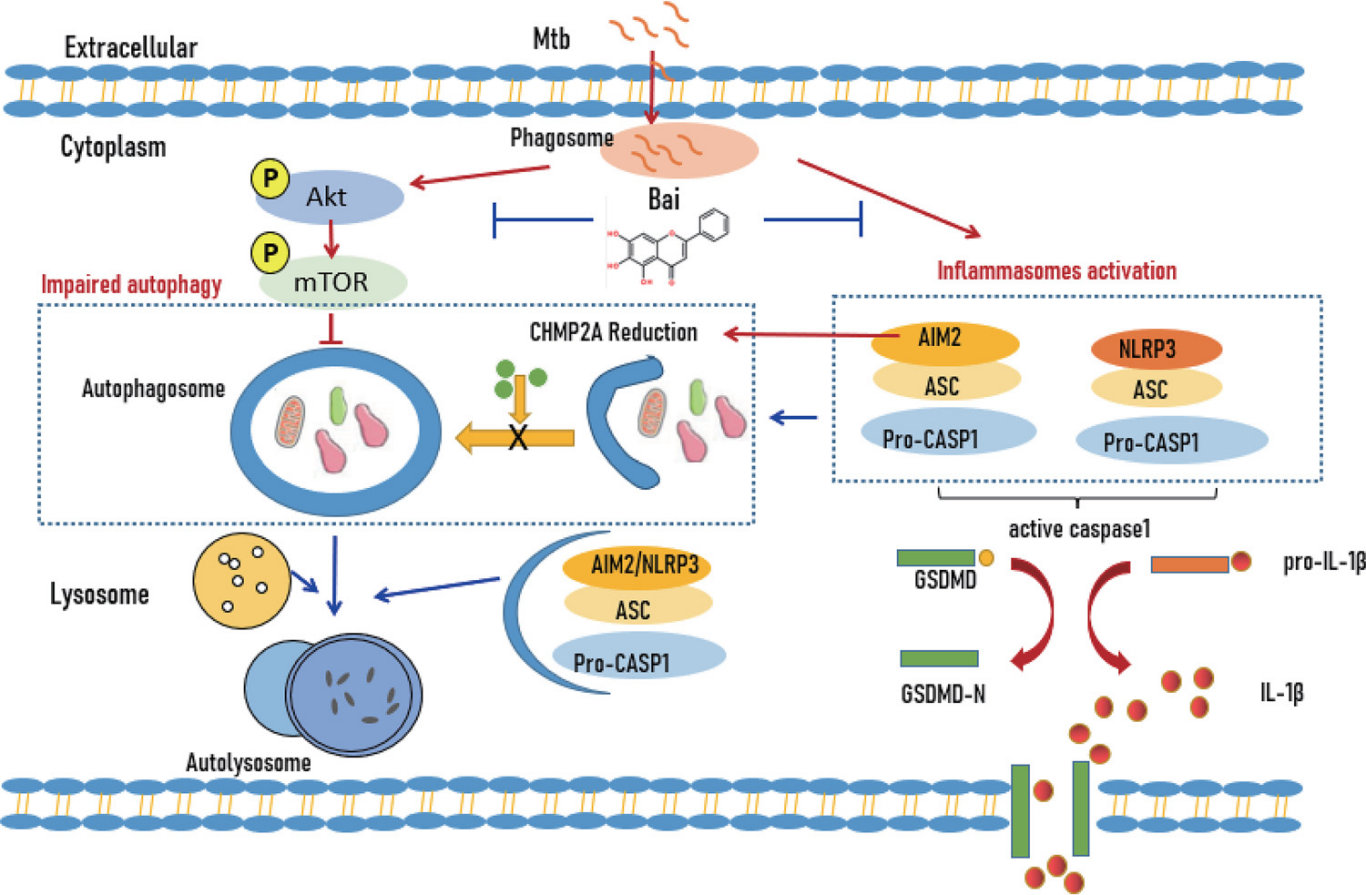
Illustration of baicalein suppressing pyroptosis in Mtb-infected macrophages via induced autophagy. Baicalein attenuated pyroptosis in Mtb-infected macrophages by inhibiting Akt-mTOR pathway activation, as well as inhibiting AIM2 to regulate CHMP2A protein to promote autophagy.

In summary, Mtb is an intracellular pathogen that evades several host defenses against infection to persist and spread within macrophages (70). It inhibits the normal series of phagosome maturation events that occur after phagocytosis to avoid its trafficking to the antimicrobial environment of acidified phagolysosomes (61, 71, 72). Mtb simultaneously leads to inflammasome activation and pyroptosis, resulting in a series of inflammatory responses (73, 74). This study demonstrated that baicalein downregulates AIM2 and NLRP3 inflammasome assembly and activation and subsequent pyroptosis following Mtb infection. Baicalein induced autophagy by inhibiting the Akt/mTOR pathway to suppress NLRP3 and AIM2 inflammasome-mediated pyroptosis. Another interesting finding was that baicalein inhibited the levels of AIM2 and NLRP3 proteins. Inhibition of AIM2 protein could regulate CHMP2A protein, complementing the autophagy-promoting effect (Fig. 7). However, a similar effect was not seen with NLRP3. Baicalein can be used as an adjunct treatment for TB or other inflammatory diseases by regulating immune function and alleviating inflammasome-mediated pyroptosis.

## MATERIALS AND METHODS

### Reagents.

Dimethyl sulfoxide (DMSO), bovine serum albumin (BSA), and phorbol 12-myristate 13-acetate (PMA) were obtained from Sigma (St. Louis, MO). The biccinchoninic acid (BCA) protein assay kit, radioimmunoprecipitation assay (RIPA) lysis buffer, antifade mounting medium protein A/G-agarose beads, and lactate dehydrogenase (LDH) cytotoxicity assay kit were purchased from the Beyotime Institute of Biotechnology (Shanghai, China). RPMI 1640 medium and Dulbecco’s modified Eagle’s medium (DMEM) were obtained from HyClone Laboratories, Inc. (Logan, UT, USA). Middlebrook 7H10 agar and 7H9 liquid media were purchased from Difco (Detroit, MI, USA). The RiboFECT CP transfection kit was purchased from Ribo Biotechnology (Guangzhou, China). The IL-1β ELISA kit was purchased from R&D (USA). Disuccinimidyl suberate (DSS) was purchased from ThermoFisher (USA).

### Antibodies.

The following antibodies were used: anti-NLRP3 (catalog number 15101), anti-Akt (catalog number 4691), anti-phospho-Akt (Ser473) (catalog number 4060), anti-LC3B (catalog number 2775), anti-IL-1β (catalog number 12242), anti-GSDMD (catalog number 39754), anti-cleaved-caspase-1 (Asp296) (catalog number 89332), anti-cleaved-caspase-1 (Asp297) (catalog number 4199), anti-phospho-mTOR (ser2448) (catalog number 5336), and anti-mTOR (catalog number 2972) were purchased from Cell Signaling Technology, Inc. (Danvers, MA, USA); anti-p62 (catalog number A11250), Alexa Fluor 488 goat anti-rabbit IgG (AS053), and anti-AIM2 (catalog number A3356) were obtained from ABclonal Technology (Wuhan, China); antibodies against ASC (catalog number sc-514414) was purchased from Santa Cruz Biotechnology, Inc. (Santa Cruz, CA, USA); and anti-β-actin (catalog number 66009-1-lg) monoclonal antibody and caspase-1 (catalog number 22915-1-AP), and anti-CHMP2A (catalog number 10477-1-AP) were acquired from Protein Tech Group (Chicago, IL).

### Drugs.

Baicalein (molecular weight, 270.237; purity > 98%; chemical structure shown in Fig. 1A) was purchased from Shanghai Tauto Biotech Co., Ltd. (CAS 491-67-8; Shanghai, China). It was first dissolved in DMSO to a storage concentration of 100 mM and kept at −20°C before being diluted in DMEM containing 10% FBS to 25, 50, and 100 M for the subsequent studies. Chloroquine (molecular weight, 319.87; purity > 98%) was purchased from Beijing Solarbio Science & Technology Co., Ltd. (CAS 54-05-7; Beijing, China). Rapamycin (molecular weight, 914.18; purity > 98%) was purchased from Beijing Solarbio Science & Technology Co., Ltd. (CAS 53123-88-9) (Beijing, China).

### Cell culture.

Human acute monocytic leukemia cells, U937 cells were maintained in RPMI 1640 medium supplemented with 10% fetal bovine serum and 1% penicillin-streptomycin at 37°C and 5% CO_2_. For experiments, U937 cells were differentiated for about 20 h with 100 nM phorbol-12-myristate-13-acetate (PMA) the day before stimulation and planted in 6-well culture plates. The medium containing PMA was replaced with PMA-free fresh completed medium, and then the macrophages were applied in subsequent experiments. The J774A.1 murine macrophage cell line was cultured in DMEM supplemented with 10% FBS in 5% CO_2_ at 37°C.

### Bacterial strains.

The Mtb H37Ra was used in this study. Middlebrook 7H9 or 7H10 broth supplemented with 0.2% glycerol, 0.05% Tween 80, and 10% Middlebrook OADC supplement was used to grow the H37Ra strain.

### Mtb infection.

J774A.1 or U937 cells were seeded at various specifications of the cell culture plates and grown at 37°C overnight. The cells (the density of J774A.1 cells was 3 × 10^6^, 6 × 10^5^, or 8 × 10^5^; the density of U937 cells was 5 × 10^6^, 8 × 10^5^, or 1 × 10^6^) were infected with Mtb H37Ra at a multiplicity of infection (MOI) of 10:1. U937 was pretreated with PMA and then infected with Mtb H37Ra. After 4 h of coincubation at 37°C, the cells were washed three times with sterile phosphate-buffered saline (PBS) and cultured with fresh RPMI 1640 or DMEM medium containing 10% FBS in the presence or absence of various concentrations of baicalein (25, 50, and 100 μM) for different times (6, 12, and 24 h).

### Measuring cell viability applying MTT assay.

Cell viability was often assessed using the tetrazolium dye 3-[4,5-dimethylthiazol-2-yl]-2,5-diphenyltetrazolium bromide (MTT). The J774A.1 (1.5 × 10^4^ cells/well) cells or U937 (2 × 10^5^ cells/well) cells were seeded into 96-well culture plates overnight. The culture medium was substituted with the medium containing different concentrations of baicalein (0, 6.25, 12.5, 25, 50, and 100 μM) for 24 h, 48 h, and 72 h, respectively. Each well received 50 μL of 5 mg/mL MTT in PBS before another 4 h of culture time. After that, the media were evacuated, and to lyse the cells, the formazan precipitate was dissolved in 150 μL of DMSO. The absorbance was determined at 570 nm with 630 nm employing a Synergy 2 Microplate Reader (Bio-Tek, USA).

### Western blotting assay.

Cells were collected and lysed in lysis buffer. The protein in the whole-cell lysate was separated by sodium dodecyl sulfate-polyacrylamide gel electrophoresis (SDS-PAGE) and further transferred onto nitrocellulose membranes (Pall, USA). After blocking with TBST (0.5% Tween 20) containing 5% (wt/vol) nonfat milk, the membranes were incubated with specific primary antibodies against GSDMD (1:1,000), NLRP3 (1:1,000), LC3B (1:1,000), p62 (1:1,000), Akt (1:1,000), p-Akt (1:1,000), mTOR (1:1,000), p-mTOR (1:1,000), ASC (1:1,000), CHMP2A (1:1,000), cleaved-caspase-1 (1:1,000), AIM2 (1:1,000), and β-actin (1:5,000) at 4°C overnight in blocking solution. Following three TBST washes, the membranes were incubated with horseradish peroxidase (HRP)-conjugated secondary antibodies (1:10,000) at room temperature for 1 h. Chemiluminescence was tested with ECL chemiluminescent kit (Thermo Scientific). The quantification of each protein was performed by ImageJ software (U.S. National Institutes of Health, Bethesda, MD, USA).

### LDH release assay.

Cell death was evaluated applying the lactate dehydrogenase (LDH) cytotoxicity assay kit. The J774A.1 or U937 cells were seeded at 24-well plates. After baicalein (50 μM) treatment for 24, 48, and 72 h, the chromogenic reagent provided in the LDH assay kit was then added, and the luminescence signals were measured using a microplate spectrophotometer. The relative amount of LDH was calculated according to the following formula: LDH relative release amount (%) = (absorbance of treated sample − absorbance of control hole of the sample)/(absorbance of maximum enzyme activity of cells − absorbance of control hole of sample) × 100.

### Coimmunoprecipitation (Co-IP).

J774A.1 and U937 cells were lysed at 4°C in ice-cold cell lysis buffer, and cell lysates were cleared by centrifugation (12,000 rpm,15 min). BCA assay was used to assess the concentrations of proteins in the supernatant. Before immunoprecipitation, samples containing equal amounts of protein were precleared with specific antibodies (3 to 4 mg/mL) overnight at 4°C with gentle rotation and then incubated with protein A/G-agarose/Sepharose beads at 4°C with gentle rotation for 3 h. Then, agarose/Sepharose beads were washed extensively with lysis buffer four times, and proteins were eluted by boiling in 1× SDS buffer before SDS-PAGE electrophoresis. Samples were boiled for 10 min and analyzed by Western blotting.

### IL-1β ELISA.

Levels of IL-1β in the culture supernatants were quantified according to the instruction manual provided with ELISA kits, purchased from BD Biosciences (San Jose, CA, USA).

### ASC oligomer cross-linking.

J774A.1 or U937 cells were seeded in 6-well plates. After the indicated treatments, the cells were lysed with cold PBS containing 0.5% Triton X-100, and the cell lysates were centrifuged at 6,000 × *g* for 15 min at 4°C. The pellets were washed twice in PBS before being resuspended in 200 μL of PBS. 2 mM DSS was added to the resuspended pellets, and the suspension was incubated at room temperature for 30 min with rotation (75). The cross-linked pellets were spun down at 6,000 × *g* for 15 min at 4°C and redissolved in 30 μL of 1× SDS-PAGE sample loading buffer. The samples were boiled for 10 min and analyzed by Western blotting.

### RNA quantification and real-time PCR.

The J774A.1 cells or U937 cells were seeded at 6-well cell culture plates and grown at 37°C overnight. The cells were infected with Mtb H37Ra (MOI = 10: 1). Then, the cells were washed three times with sterile PBS after 4 h and cultured with DMEM or RPMI 1640 containing 10% FBS in the presence and absence of baicalein (50 μM) for 6, 12, and 24 h. The total RNA was extracted with EZ-press RNA purification kit according to the manufacturer’s instruction. The mRNAs were reverse transcribed using PrimeScript RT reagent kit (TaKaRa, RR036A). Real-time quantitative PCR analysis was performed by the SYBR PCR kits. The relative expression level of mRNAs was normalized to that of internal control β-actin/GAPDH, respectively, by using a 2^−ΔΔCt^ cycle threshold method. The following primers were used: GSDMD (mouse) forward, 5′-ATGCCATCGGCCTTTGAGAAA-3′; GSDMD (mouse) reverse, 5′-AGGCTGTCCACCGGAATGA-3′; NLRP3 (mouse) forward, 5′-GCTGCGATCAACAGGCGAGAC-3′; NLRP3 (mouse) reverse, 5′-CCATCCACTCTTCTTCAAGGCTGTC-3′; AIM2 (mouse) forward, 5′-TGAAAACTGCTCTGCTGCCTCTG-3′; AIM2 (mouse) reverse, 5′-GCTTCCTGTTCTGCCACCATCTG-3′; β-actin (mouse) forward, 5′-AGCCATGTACGTAGCCATCC-3′; β-actin (mouse) reverse, 5′-TCTCAGCTGTGGTGGTGAAG-3′; GSDMD (human) forward, 5′-GCCTCCACAACTTCCTGACAGATG-3′; GSDMD (human) reverse, 5′-GGTCTCCACCTCTGCCCGTAG-3′; NLRP3 (human) forward, 5′-CTTGCCGACGATGCCTTCCTG-3′; NLRP3 (human) reverse, 5′-GCTGTCATTGTCCTGGTGTCTTC-3′; AIM2 (human) forward, 5′-CCAATTCAAGCCAACTGGTCTAAGC-3′; AIM2 (human) reverse, 5′-TGGAGAGAGGAGCCTGTGAACTG-3′; GAPDH (human) forward, 5′-TCGACAGTCAGCCGCATCTTCTTT-3′; and GAPDH (human) forward, 5′-ACCAAATCCGTTGACTCCGACCTT-3′.

### siRNA-mediated gene silences in J774A.1 cells.

J774A.1 cells were plated in 6-well plates (at a density of 2.5 × 10^5^ cells/well) and then were transfected with 20 μM siRNA according to the manufacturer’s guidelines. GenePharma chemically synthesized siRNA sequences, and the negative-control siRNA was also from GenePharma. The siRNA sequences were as follows: siAIM2, 5′-CGCACAGUUUAAAGAUAAATTUUUAUCUUUAAACUGUGCGTT-3; siNLRP3, 5′-GGCGAGACCUCUGGGAAAATTUUUUCCCAGAGGUCUCGCCTT-3′; and negative control, 5′-UUCUCCGAACGUGUCACGUTT-3′ and 5′-ACGUGACACGUUCGGAGAATT-3′.

### Immunofluorescence.

The J774A.1 cells were seeded in special plates for immunofluorescence and incubated overnight at 37°C. The next day, Mtb H37Ra was added after the cells adhered to the wall. After 4 h of treatment, the cells were washed away with sterile PBS. After 12 h of drug treatment, we followed the steps of paraformaldehyde fixation, permeabilization, closure, primary antibody, washing, fluorescent secondary antibody, washing, staining 4′,6-diamidino-2-phenylindole (DAPI), washing, and finally adding 1 mL PBS in sequence. The steps after the secondary antibody need to be done with care to avoid light. The localization of LC3 should be observed under a confocal microscope.

### Statistical analysis.

The results were analyzed using GraphPad Prism 8 (GraphPad Software, La Jolla, CA, USA). *P* values were evaluated by employing one-way analysis of variance (ANOVA). The data shown are representative of at least triplicate experiments. A value of *P* < 0.05 was considered to be statistically significant.
